# Sex determines cortisol and alpha‐amylase responses to acute physical and psychosocial stress in patients with avoidant personality disorder

**DOI:** 10.1002/brb3.506

**Published:** 2016-06-14

**Authors:** Yoshihiro Tanaka, Yoshinobu Ishitobi, Ayako Inoue, Harumi Oshita, Kana Okamoto, Chiwa Kawashima, Mari Nakanishi, Saeko Aizawa, Koji Masuda, Yoshihiro Maruyama, Haruka Higuma, Masayuki Kanehisa, Taiga Ninomiya, Jotaro Akiyoshi

**Affiliations:** ^1^ Department of NeuropsychiatryOita University Faculty of MedicineHasama‐MachiOita879‐5593Japan; ^2^ Department of Applied LinguisticsOita University Faculty of MedicineHasama‐MachiOita879‐5593Japan

**Keywords:** Alpha‐amylase, avoidant personality disorder, cognition, cortisol, social anxiety disorder, stress

## Abstract

**Introductions:**

Avoidant personality disorder (AVPD) has excessive and pervasive anxiety and discomfort in social situations. The aims of this study were to explore the relationship between AVPD and physical and psychological stress and psychological tests.

**Methods:**

We evaluated 93 AVPD patients and 355 nonpatient controls by salivary amylase and cortisol responses during exposure to the Trier Social Stress Test (TSST) and electrical stimulation stress. Spielberger state‐trait anxiety inventory (STAI), Profile of Mood State (POMS), Beck Depression Inventory (BDI), Depression and Anxiety Cognition Scale (DACS), and Childhood Trauma Questionnaire (CTQ) were administered.

**Results:**

Following electrical stimulation, salivary cortisol levels in female AVPD decreased significantly less than that in female's controls, but salivary cortisol levels did not show a difference between male AVPD patients and controls. Salivary alpha‐amylase (sAA) levels did not show a difference between females or male AVPD patients and controls. Following TSST exposure, sAA levels did not show a difference between females or male AVPD patients and controls. Salivary cortisol levels did not show a difference between females or male AVPD patients and controls. In the AVPD patients, POMS scores were significantly higher compared with the controls. STAI, BDI, DACS scores, and CTQ significantly increased in the AVPD patients compared with the controls. LF in heart rate variability in AVPD significantly increased more compared with controls.

**Conclusions:**

These results suggest that heightened sympathetic reactivity in female AVPD co‐occurs with attenuated salivary cortisol responses to electric stimulation stress and there is a significant difference between AVPD and controls in mood, anxiety, social cognition, and automatic nerve systems.

## Introduction

Avoidant personality disorder (AVPD) is one of the most common personality disorders that lead to serious and chronic impairment of the interpersonal and occupational function (Weinbrecht et al. 2016). This disorder is characterized by a pattern of avoidance of interpersonal contact due to fear of criticism, disapproval, or rejection (DSM‐V; APA, 2013). Severe disturbance in interpersonal relationships lead to significant limitations in the ability to function in the workplace. Anticipation of exposure to social situations increases the anxiety level of AVPD patients.

Avoidant personality disorder shares many characteristics with social anxiety disorder (SAD). AVPD and SAD in the DSM system have been diagnosed as a separate disease. It is defined by “The most apparent overlap of SAD is with AVPD. Individuals with AVPD have a broader avoidance pattern than those with SAD. Nonetheless, SAD is typically more comorbid with AVPD than with other personality disorders, and AVPD is more comorbid with SAD than with other anxiety disorders” (DSM‐V; APA, 2013; p. 207). These symptoms include (1) avoiding interaction with many people for fear of criticism and (2) preoccupation with being criticized or rejected in the context of interpersonal exchanges. There is a possibility that more than any of the boundary values for social anxiety between two of SAD and AVPD (Tillfors and Ekselius 2009). AVPD could influence the course of SAD in adulthood (Cox et al. 2011). Although AVPD and SAD have a lot of common symptoms, AVPD has some unique symptoms that are not found by SAD diagnosis (Marques et al. 2012). AVPD and SAD may have different risk factors which may be influenced to by heritable traits (Torvik et al. 2015; Lampe 2016).

Personal variation in personality traits was associated with the neural response to mental stress (Yamano et al. 2015). The stress reaction is mainly regulated by an axial system consisting of two neuroendocrine systems: the hypothalamic–pituitary–adrenocortical (HPA) axis and sympathetic adrenomedullary system. A treatment combining virtual reality exposure therapy and administration yohimbine hydrochloride leads to a significant reduction in anxiety compared with pretreatment induces significantly higher levels of salivary alpha‐amylase (sAA) in the treated group (Meyerbroeker et al. 2012). While self‐reported anxiety increased in patients with SAD before, during, and after Trier Social Stress Test (TSST), differences in the biological stress response (sAA, HR, and heart rate variability [HRV]) were not observed between the SAD and healthy controls (Klumbies et al. 2014). In another study, there were no significant differences between SAD subjects and controls in terms of TSST (Martel et al. 1999). To date, there are no reports about the relationship between AVPD, and sAA or salivary cortisol levels.

Decreased HRV response to task performance (writing mode of random number generation) is related to anxiety. Decreased autonomic responsiveness could provide as a sign of psychological dysfunction (Shinba et al. 2008). The information processing in cognitive avoidant coping (spatial cueing paradigm with emotional and neutral facial expressions as cues) is related to a decrease in HRV in response (Schwerdtfeger and Derakshan 2010). There are no studies to exam the relationship between AVPD and HRV.

The patients with AVPD might be too exhausted by their anxiety (Weinbrecht et al. 2016). Recently we reported that Depression and Anxiety Cognition Scale (DACS) scores showing significant interaction with the two SNPs for microcephalin 1, syntrophin‐beta 1 may be regarded as appropriate traits to detect the diathesis of automatic thoughts (Ishitobi et al. 2014) and DACS scores were significantly increased in borderline personality disorder patient compared with controls (Inoue et al. 2015). These automatic thoughts might proceed as mediators between schemas and prospective changes in AVPD. Some reports suggested that AVPD was associated with child abuse (Vrabel et al. 2010; Waxman et al. 2014). It is important to study for relationship of between AVPD and child abuse. Kampmann et al. (2016) reported that anxiety was concerned with AVPD in social interaction. Though the mechanism of AVPD has not been clearly defined, neglect and rejection in childhood are associated with an increased risk for the development of AVPD (Joyce et al. 2003; Eggum et al. 2009).

Some reports reported that AVPD was more and traits among females (Zimmerman and Coryell 1989; Ullrich and Coid 2009; Grant et al. 2012).

We hypothesized that sex determines salivary cortisol and sAA responses to different stress in patients with AVPD. In this study, we investigated stress reactivity in AVPD and its interference with social cognition.

## Methods

### Subjects

Ninety‐three subjects meeting the Diagnostic and Statistical Manual of Mental Disorders (DSM)‐IV‐TR criteria for AVPD and 355 healthy control subjects participated in the study. They were a Japanese population. Subjects presenting features of AVPD were recruited via advertisement and were screened by an interview. Subjects who fitted DSM‐IV‐TR criteria for AVPD and who did not have axis I disorders were selected in the study. The diagnosis of AVPD was determined using the Structured Clinical Interview of DSM‐IV‐TR for Personality Disorders (SCID‐II) Personality Questionnaire (First et al. 1997). The diagnoses of current Axis I disorders were made by a trained psychiatrist (JA) using the Mini International Neuropsychiatry Interview (MINI), a standardized psychiatric examination validated in the general population (Sheehan et al. 1998) according to the DSM‐IV‐TR criteria (Ritchie et al. 2004). We excluded 15 AVPD patients with comorbid axis I disorder (eight major depressive disorder [MDD], five panic disorder, and two bipolar disorder). Healthy control participants were enrolled via advertising and needed to be free of any axis I or II disorder (as determined by MINI and SCID‐II). All participants were free of major medical illnesses according to medical history and physical examination and did not show any substance or alcohol abuse or dependence within 12 months prior to the study. The participants did not take psychotropic medication for at least 12 weeks prior to the beginning of the study.

Demographic information (age, education) was collected from all participants. The participants were instructed to avoid strenuous physical activity 48 h and any form of physical exercise, as well as alcohol consumption, 24 h before the experiments. Caffeine, tea, and smoking were not permitted within 3 h prior to the study, and tooth brushing or eating was to be avoided 2 h avoid before the study. To diminish the influence of circadian rhythm on physiological variables, all experiments were performed in the afternoon (between 1 and 5 p.m.). All female subjects participated in the experiment during their late luteal phase to reduce the impact of hormonal variations through the menstrual cycle. After receiving a comprehensive explanation of the study, the participants offered written informed consent for participation. The study was approved by the ethics committees of the Oita University Faculty of Medicine.

### Stimuli and procedures

#### Stress challenge

All subjects were exposed to both TSST and electrical stimulation stress on separate days within 1 week. To exclude the effect of being habituated and relaxed to the experimental environment, the participants were divided into small groups consisting of four to five subjects. Each participant was assigned to undergo both experiment of electric stimulation and TSST. The second trial's experiment was administered 1 week after the first experiment.

#### Electrical stimulation test

All participants were invited to our laboratory on a weekday afternoon between 1 and 5 p.m. (Ishitobi et al. 2010; Maruyama et al., 2012; Tanaka et al. 2012a,b; Kawano et al. 2013; Tamura et al. 2013; Tanaka et al. 2013). The subjects wore stimulator coils on the right wrist; these coils were connected to a stimulator that provided an electrical current to the motor and sensory fibers of the median nerve. The change (variation) of heart rate during short term (5 min) was analyzed with the method of time domain and frequency domain to provide the degree of balance and activity of autonomic nervous system. We put the finger sensor into the right forefinger. The subjects received stimuli in incremental steps until they reached their threshold stimulus, which was defined as the greatest stimulus they could tolerate. The threshold stimulus for each subject was applied for 40 sec. The mean intensity of electrical stimulation was between 24.6 ± 13.1 mA in controls and 22.6 ± 12.4 mA in AVP. The range of the electrical stimulation was 2–55 mA. Thus, the magnitude of electrical stimulation experimentally varied across subjects. The subjects were told that the level of electrical stimulation would be sufficient to cause pain but would not cause burning or other injury. The electrical stimulation lasted only briefly and did not cause any physical impairment. The intensity applied appeared to reflect individual sensitivities. The threshold of electrical stimulation may be dependent on interindividual differences related to psychological factors such as depression and anxiety (Tanaka et al. 2012a,b). Moreover, the strength of the stimulation was determined by each subject. Therefore, this pain‐causing experimental setting was not contrary to the ethics of human experimentation.

#### Trier Social Stress Test

All participants were invited to our laboratory on a weekday afternoon between 1 and 5 p.m. After a 30‐min resting period to minimize the impact of physical activity, prior stress, and emotions, during which all participants filled out some questionnaires, they were exposed to TSST (Kirschbaum et al. 1993). TSST consists of a 3‐min preparation period, a 5‐min speech task, during which the participants had to discourse about their personal characteristics, and a 5‐min mental arithmetic task. Both tasks were performed in front of an audience. After the test, the participants remained in our laboratory for another 20 min for a collection of samples during recovery.

### Psychological measures

The Profile of Mood States (POMS), the Spielberger Anxiety Inventory (STAI), Beck Depression Inventory (BDI) scores, and DACS were administered to the participants before the electrical stimulation protocol (Spielberger et al. 1973; McNair et al. 1992; Fukui 1998). A psychiatrist conducted a medical examination and assessed the severity of social anxiety symptoms using the clinician administered Liebowitz Social Anxiety Scale (LSAS; Fresco et al. 2001). POMS assessment provides a rapid, economical method of assessing transient, fluctuating active mood states. It is an ideal instrument for measuring and monitoring treatment change in clinical, medical, and addiction counseling centers. STAI clearly differentiates between the temporary condition of “state anxiety” and the more general and long‐standing quality of “trait anxiety” and helps professionals distinguish between a client's feelings of anxiety and depression.

### Physiological measures

We measured sAA and salivary cortisol levels before, immediately after, and 20 min after electrical stimulation or TSST as in previous reports (Ishitobi et al. 2010; Maruyama et al., 2012; Tanaka et al. 2012a,b; Kawano et al. 2013; Tamura et al. 2013; Tanaka et al. 2013). To control for circadian variations in sAA and cortisol levels, the exposure to physical stressors and collection of saliva were performed between 1 and 5 p.m. The levels of sAA were measured using the Dry Chemistry System (Nipro Corp., Osaka, Japan) according to the manufacturer's protocol. Saliva was sampled by directly immersing a saliva‐sampling strip in saliva under the tongue for 30 sec. The strip was immediately placed in an automatic saliva transfer system, and saliva was transferred to the alpha‐amylase test paper on the reverse side of the strip sleeve by compression. The alpha‐amylase test paper contained the substrate 2‐chloro‐4‐nitrophenyl‐4‐*O*‐*β*‐d‐galactopyranosylmaltoside. The enzyme reaction started upon transfer by compression, and the level of free 2‐chloro‐4‐nitrophenyl was optically measured after 20 sec. The alpha‐amylase activity that reduced sugars equivalent to 1 μmol/min of maltose was defined as 1 unit (Robles et al. 2011). The concentration of salivary cortisol (μg/dL) was analyzed by enzyme‐linked immunosorbent assay, with intraassay and interassay coefficients of variation in 3 and 10%, respectively. The samples were stored in a freezer at −20°C until they were thawed for analysis. To evaluate the responsiveness over time, the area under the curve with respect to increase (AUCi) was calculated for both sAA and salivary cortisol levels according to Pruessner et al. (2003).

We also measured HRV immediately after the electrical stimulation. Low frequency (LF; 0.04–0.15 Hz) and high frequency (HF; 0.15–0.4 Hz) fluctuations of HR on R–R intervals were calculated by an APG Heart‐Rater SA‐3000P (Tokyo Iken Co, Ltd, Tokyo, Japan). The LF component had been frequently used in the past as a measure of sympathetic nervous system activity, but recent pharmacological research has repeatedly questioned its validity, indicating that LF power of HRV is not a measure of cardiac sympathetic tone but may be a measure of the modulation of cardiac autonomic outflows by baroreflexes (Goldstein et al. 2011). In particular HF fluctuations (also referred to as respiratory sinus arrhythmia) are highly sensitive to confounding influences such as respiratory parameters (tidal volume) and other factors (participant's level of fitness, medication, and position at assessment). Without the assessment of these confounders, it is not possible to perform valid HF calculations. We also added the total power (TP), standard deviation of the NN intervals (SDNN), and root mean square of successive differences (RMSSD).

### Statistical analyses

We adjusted all analyses to match age. Three hundred and fifty‐five volunteers were selected to provide close matches to the 93 patients with respect to age and gender. The data are presented as the mean ± standard deviation (SD) and reliability coefficients (Cronbach's alpha) of the individual values from each test. We performed analyses using the SPSS 19 software (SPSS Inc., Chicago, IL). We used the *χ* ^2^ test, Mann–Whitney's *U* test, and Spearman rank‐correlation coefficient for sample characterization. Repeated measures analysis of variance was used to compare sAA or cortisol response level means between the patients and controls followed by Scheffe's significant difference tests. Results were analyzed structural equation modeling (SEM) using SPSS AMOS 21.0 (SPSS Inc.). We used comparative fit index as the model. Statistical significance was set at *P *<* *0.05.

## Results

### Psychological assessment

**Figure 1:**
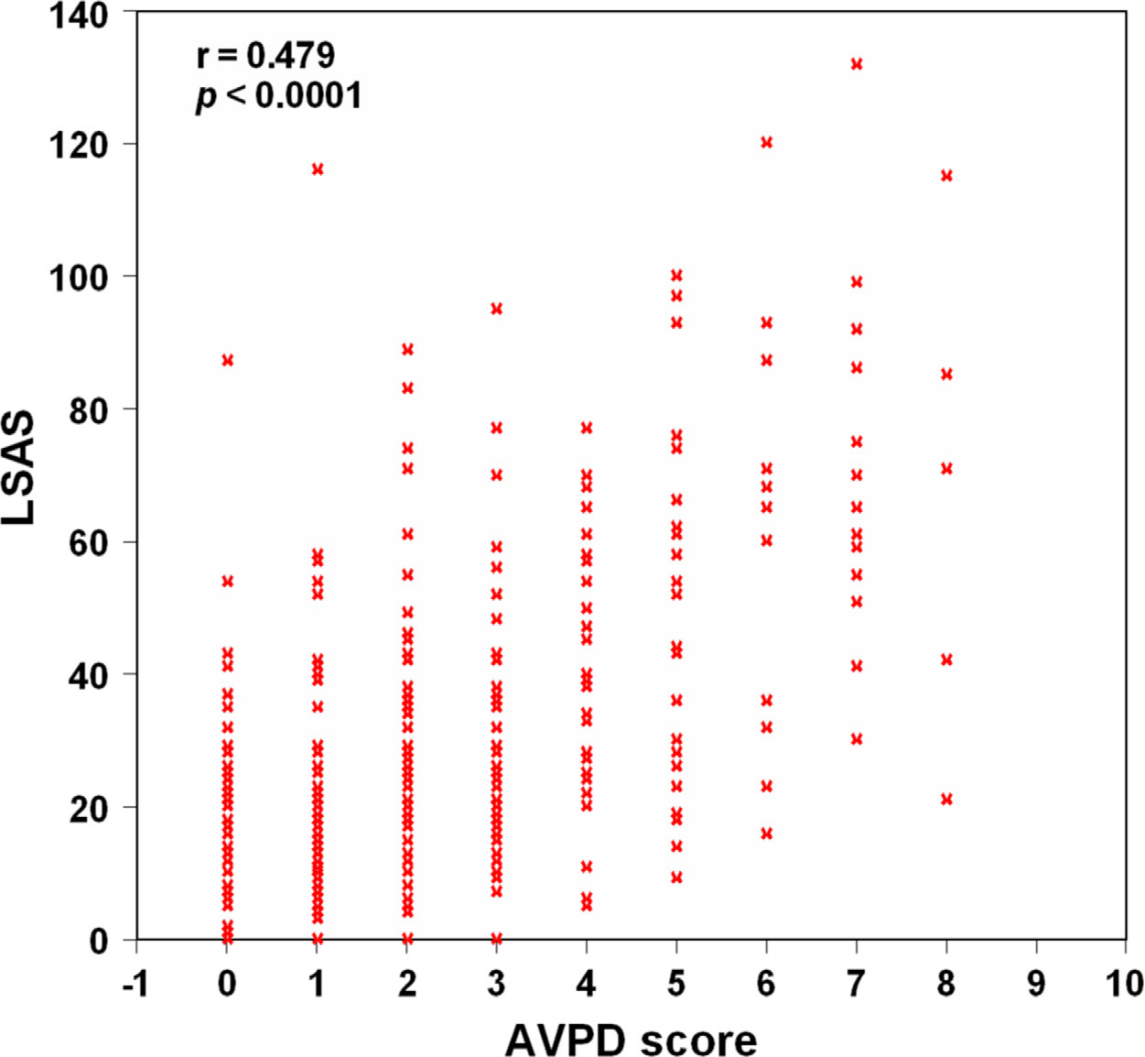
The relationship between Liebowitz Social Anxiety Scale (LSAS) and avoidant personality disorder (AVPD) score on SCID‐II.

There was no difference between the AVPD and healthy groups with regard to age, smoking history, and education level, but the two groups were significantly different in terms of gender distribution (Table 1). Regarding POMS testing, the AVPD patients scored significantly higher across five categories (tension–anxiety, depression–dejection, anger–hostility, fatigue, and confusion) and significantly lower in the vigor category compared with the controls (*P *<* *0.01) (Table S1). STAI scores and BDI scores were significantly higher in the AVPD patients compared with the controls (*P *<* *0.01) (Table S1). DACS scores (future denial, threat prediction, self‐denial, past denial, and interpersonal threat) were significantly increased in AVP patient compared with controls (*P *<* *0.01). Childhood Trauma Questionnaire (CTQ; emotional abuse, sexual abuse, emotional neglect, physical neglect) were significantly increased in AVPD patient compared with controls. The LSAS was significantly correlated with AVPD score on SCID‐II (Fig. 1). The reliability coefficient (Cronbach's alpha) of the POMS, STAI, DACS, and CTQ for the samples was 0.78, 0.82, 0.79, and 0.73.

**Table 1:** Demographic and medical characteristics by group

	Control	AVP	*P* or *χ* ^2^
*N*	355	93	
Age (years)	24.9 ± 3.9 (22–51)	25.0 ± 3.6 (22–36)	0.83
Sex (female/male)	141/214	40/53	<0.0001
Smoking (no. cig)			
<10/day	327	86	
>10/day	28	7	*χ* ^2^ = 0.01; *P *=* *0.91
Education (years)	13.7 ± 2.0	13.6 ± 2.0	*P *=* *0.68

AVP, avoidant personality disorder; BDI, Beck Depression Inventory.

### Physiological assessment

**Figure 2:**
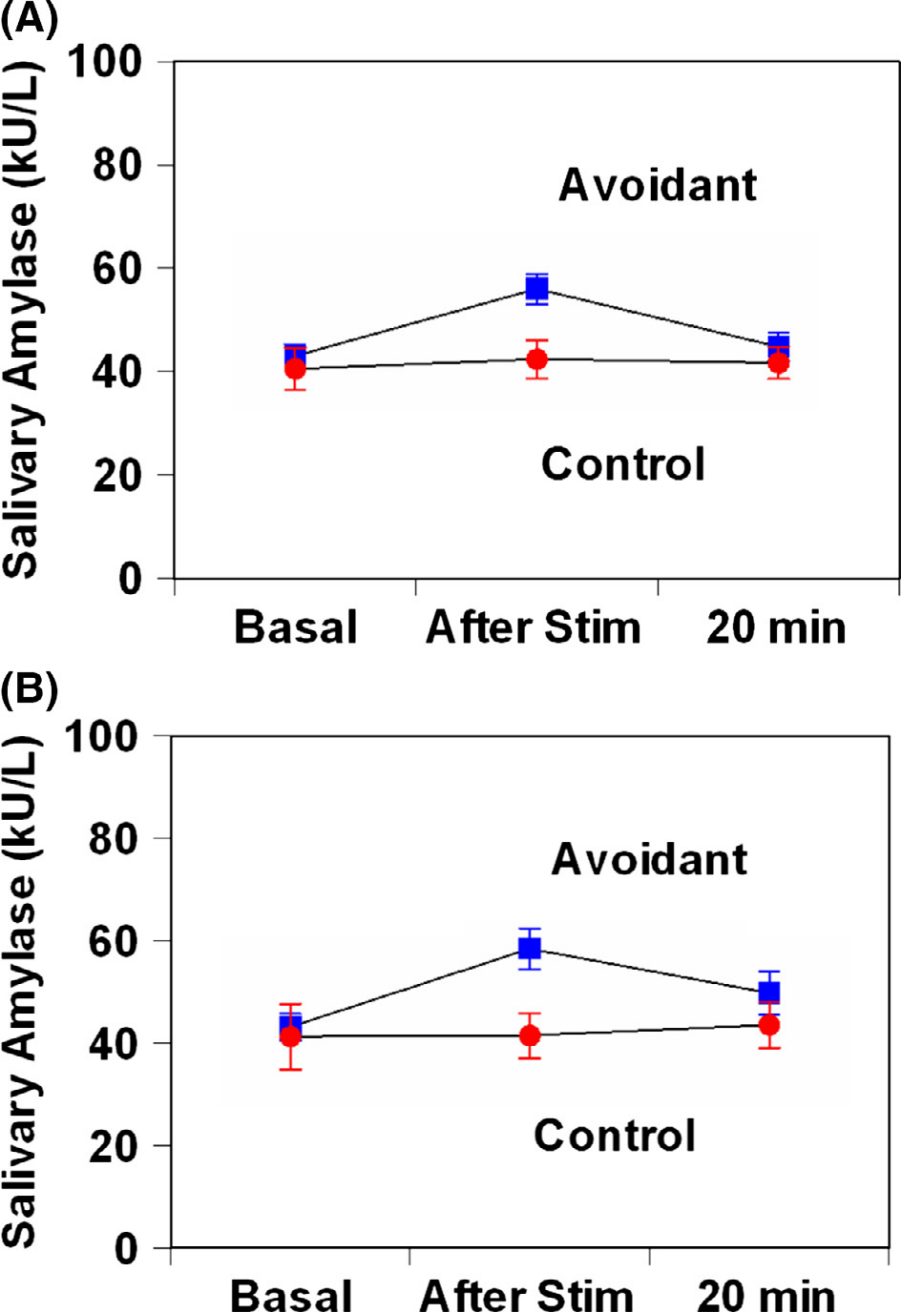
Salivary alpha‐amylase responses to Trier Social Stress Test in patients with avoidant personality disorder and healthy matched control subjects. (A) Female; (B) male. Values are presented as mean ± standard deviation.

**Figure 3:**
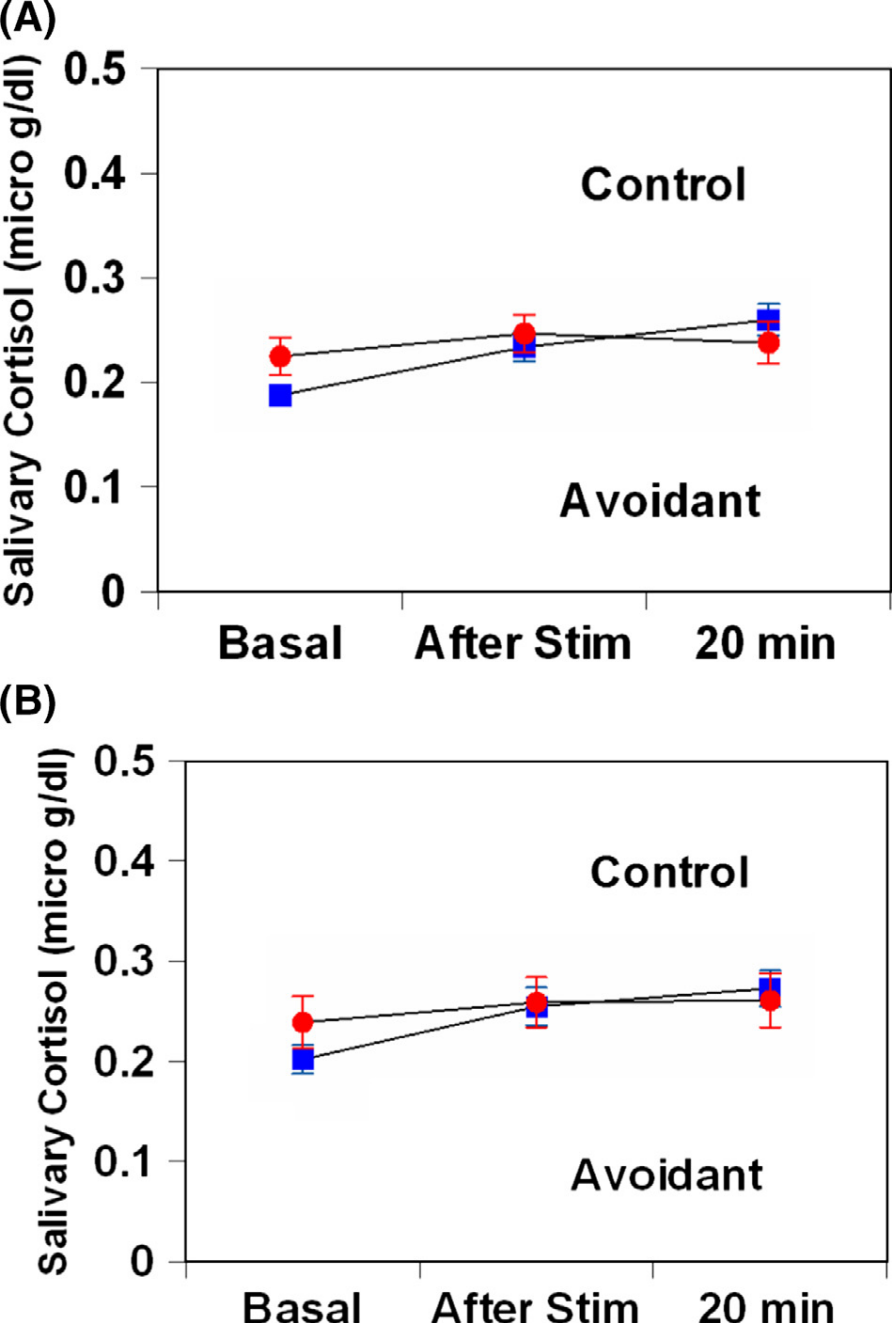
Salivary cortisol responses to Trier Social Stress Test in patients with avoidant personality disorder and healthy matched control subjects. (A) Female, (B) male. Values are presented as mean ± standard deviation.

Following TSST exposure, sAA levels did not show a difference between females or male AVPD patients and controls (*F*_2,446_ = 2.35, *P *=* *0.13, Fig 2A; *F*_2,446_ = 2.16, *P *=* *0.14, Fig 2B). Following TSST exposure, salivary cortisol levels did not show a difference between females or male AVPD patients and controls (*F*_2,446_ = 0.18, *P *=* *0.68, Fig 3A; *F*_2,446_ = 0.11, *P *=* *0.74, Fig 3B).

**Figure 4:**
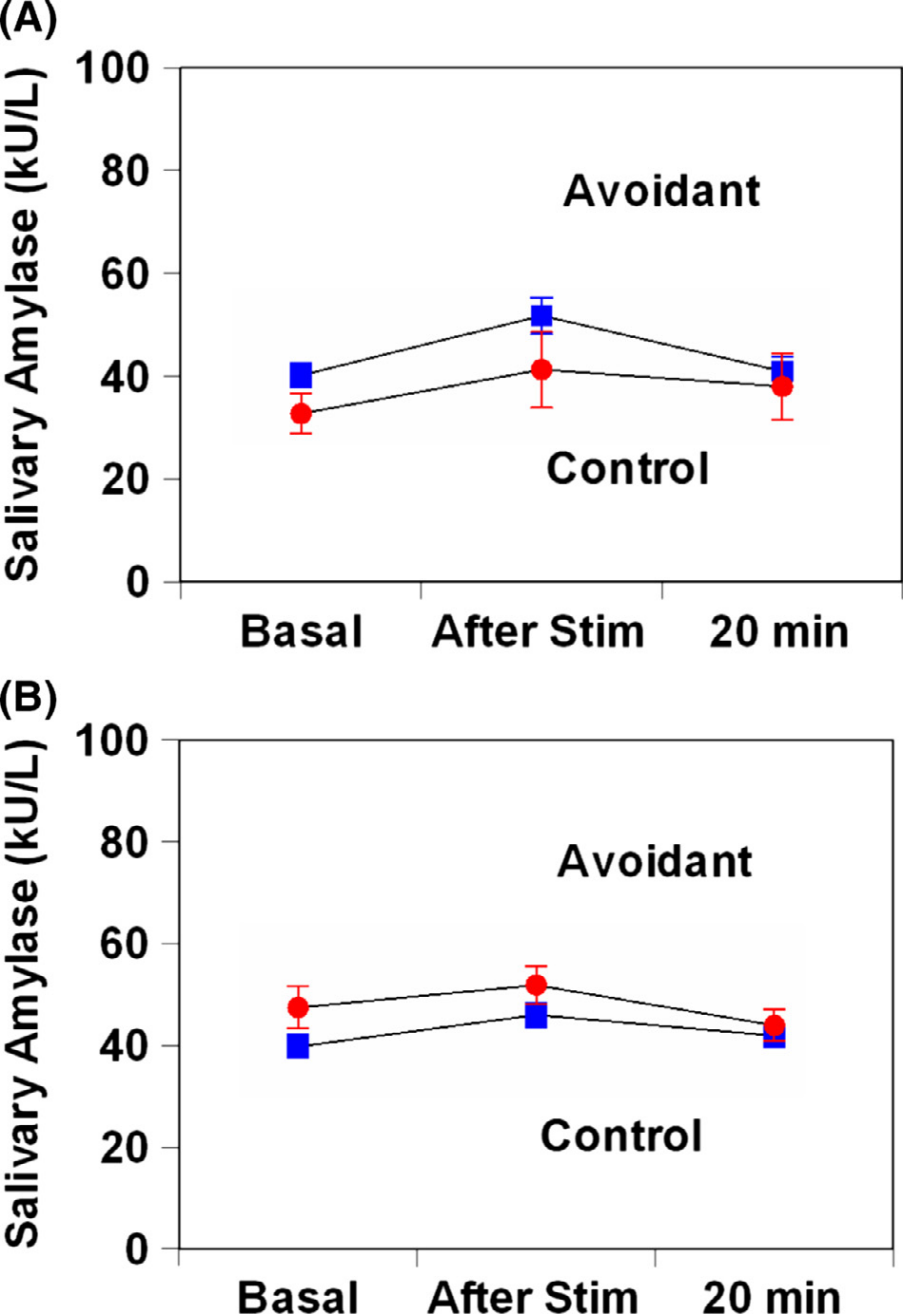
Salivary alpha‐amylase responses to electrical stimulation in patients with avoidant personality disorder and healthy matched control subjects. (A) female; (B) male. Values are presented as mean ± standard deviation.

**Figure 5:**
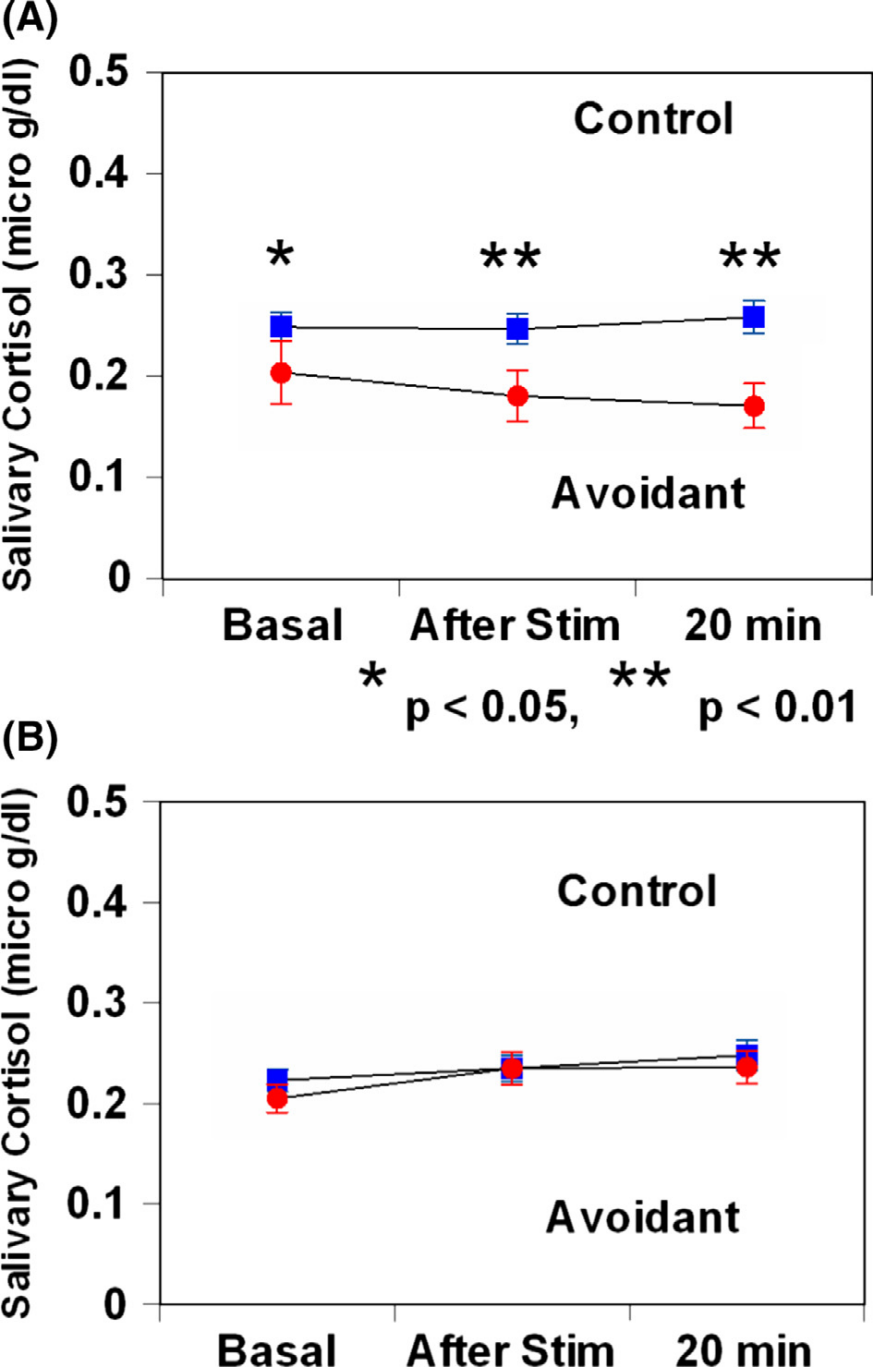
Salivary cortisol responses to electrical stimulation stress in patients with major depressive disorder and healthy matched control subjects. (A) female; (B) male. Values are presented as mean ± standard deviation. *P < 0.05. **P < 0.01.

Following electrical stimulation, sAA levels did not show a difference between females or male AVPD patients and controls (*F*_2,446_ = 1.50, *P *=* *0.22, Fig 4A; *F*_2,446_ = 2.24, *P *=* *0.14, Fig 4B). Following electrical stimulation, salivary cortisol levels in female AVPD decreased significantly less than that in females controls (*F*_2,446_ = 1.50, *P *=* *0.04, Fig 5A). On the other hand, salivary cortisol levels did not show a difference between male AVPD patients and controls (*F*_1,112_ = 0.26, *P *=* *0.61, Fig. 5B). There were no differences in sAA or salivary cortisol AUCi between female or male AVPD and controls.

There were no differences in the strength of electrical stimulation between controls and AVP patients (Table S1). There were no differences in LF/HF, TP, and SDNN between controls and AVPD patients (Table S1). LF in AVPD significantly increased more compared with controls (*P *<* *0.01). HF and RMSSD in AVPD significantly decreased less compared with controls (*P *<* *0.01, *P *<* *0.05).

The results of covariance structure analysis in the SEM from salivary cortisol response to POMS, STAI, BDI, DACS, CTQ, and HRV were not significant.

## Discussion

Heightened sympathetic reactivity in female AVPD co‐occurs with attenuated salivary cortisol responses to electric stimulation stress and there is a significant difference between AVPD and controls in mood, anxiety, social cognition, and automatic nerve systems.

Following electrical stimulation, salivary cortisol levels in female AVPD decreased significantly less than that in female controls. On the other hand, salivary cortisol levels did not show a difference between male AVPD patients and controls. Following TSST exposure, sAA and salivary cortisol levels did not show a difference between females or male AVPD patients and controls. We already reported that sAA in SAD patients displayed a significant higher level at baseline and a significantly larger response to electrical stimulation as compared to healthy controls (Tamura et al. 2013). This result is consistent with the results which in SAD there is a vulnerability of the autonomic nervous system more than the HPA axis (van Veen et al. 2009). However, the present sAA result in AVPD was not consistent with the former results in SAD (van Veen et al. 2009; Tamura et al. 2013). Recently, Torvik et al. (2015) reported that the relationship between SAD and AVPD was best explained for by a model with separate. The finding of partially separate risk factors (genetic and environmental components) indicates qualitative differences in the etiology of AVPD and SAD. Lampe (2016) also reported that AVPD and SAD distribute genetic vulnerability, but may have different environmental risk factors that form qualitative differences. Negative self‐concept, shame tendency, and interpersonal reaction are property of AVPD and may be influenced to by heritable traits of high negative and low positive affective state, and experiences of negligent or impassive parents. The negative emotional state may suppress salivary cortisol for a long time and it did not show the elevation after electrical stimulation stress as a result. These results suggest that there is a different the autonomous nervous system response to physical stress between AVPD and SAD. Further studies are needed to discriminate the differences between AVPD and SAD.

High frequency and RMSSF in AVPD significantly decreased less compared with controls. There were no differences in LF/HF between controls and AVPD patients. HF frequency was frequently used as a measure of parasympathetic nerve activity. These results is consistent with that salivary cortisol levels in female AVPD decreased significantly less than that in female controls. The present results suggested that AVPD showed lowered parasympathetic nerve activity in AVPD. However, the results are not consistent with our past report in AVPD that there were no group differences in any HRV of SAD (Tamura et al. 2013) and low baseline HF represents a common index for inhibitory deficits across SAD (Pittig et al. 2013). The SNS activity in AVPD might be different from that in SAD.

Regarding POMS testing, AVPD patients scored significantly higher across five categories (tension–anxiety, depression–dejection, anger–hostility, fatigue, and confusion) and significantly lower in the vigor category compared with controls. The present results are insistent with that in SAD (Tamura et al. 2013). STAI scores were significantly increased in AVP patients compared with controls. The present results are insistent with that in SAD (Tamura et al. 2013). These results are also consist with borderline personality disorder, obsessive‐compulsive disorder, panic disorder, and MDD (Tanaka et al. 2012a,b; Kawano et al. 2013; Inoue et al. 2015). POMS might show the same patterns for psychological stress, such as depression and anxiety.

Depression and Anxiety Cognition Scale scores (future denial, threat prediction, self‐denial, past denial, and interpersonal threat) were significantly increased in AVPD compared with controls. This result suggested the relationship between automatic thoughts and AVPD. The depressed outpatients with a personality disorder with maladaptive avoidant and paranoid beliefs showed more severe depressive symptomatology at intake and more residual symptoms at termination to cognitive therapy (Kuyken et al. 2001). Participants with avoidant symptoms consistently evaluated the scene relatively more negatively in priming state (Bowles and Meyer 2008). The therapy for AVPD is maintained at follow‐up treated with cognitive‐behavioral therapy (Simon 2009). This results are also consist with borderline personality disorder (Inoue et al. 2015). Further studies are needed to the role of cognition in AVPD.

Childhood Trauma Questionnaire (emotional abuse, sexual abuse, emotional neglect, physical neglect) significantly increased in the AVPD patients compared with the controls. The role of childhood traumatic occurrences causing to various types of PDs, such as avoidant PD (Yen et al. 2002) has been extraordinary. However, we did not show the data for different personality disorders in relation to CTQ. Further studies are need to examine the relationship between AVPD and different personality disorders in relation to CTQ.

First, the limitation of this study is that the small sample size prevents us from drawing definite conclusions. We are also limited in being unable to generalize this finding to other ethnic groups. However, our investigation was performed in a single population and it shows the superiority of this study. We consider it necessary to study another larger population for reproducibility. Second, we examined HRV (LF, HF, and LF/HF) following the electrical stimulation test but not following TSST. As there is only one measurement point for HRV, it is not correct to interpret the differences between male AVPD patients and controls as increase or decrease. It might rather reflect baseline differences. Third, we used different amplitudes of electrical stimulation according to participant tolerance. Therefore, the amplitude of electrical stimulation varied.

In conclusion, these results suggest that heightened sympathetic reactivity in female AVPD co‐occurs with attenuated salivary cortisol responses to electric stimulation stress and there is a significant difference between AVPD and controls in mood, anxiety, social cognition and automatic nerve systems.

## Conflict of Interest

None declared.

## Supporting information


**Table S1.** Characteristics of avoidant personality disorder patients and controls.
